# A different rhythm of life: sleep patterns in the first 4 years of life and associated sociodemographic characteristics in a large Brazilian birth cohort

**DOI:** 10.1016/j.sleep.2017.06.001

**Published:** 2017-09

**Authors:** Elena Netsi, Ina S. Santos, Alan Stein, Fernando C. Barros, Aluísio J.D. Barros, Alicia Matijasevich

**Affiliations:** a Department of Psychiatry, University of Oxford, Oxford, UK; b Postgraduate Program in Epidemiology, Federal University of Pelotas, Pelotas, Brazil; c MRC/Wits Rural Public Health and Health Transitions, Research Unit (Agincourt), School of Public Health, University of the Witwatersrand, Johannesburg, South Africa; d Post-Graduate Program in Health and Behavior, Catholic University of Pelotas, Pelotas, RS, Brazil; e Department of Preventive Medicine, School of Medicine, University of Sao Paulo, São Paulo, Brazil

**Keywords:** Child sleep, Pelotas 2004 Birth Cohort, Co-sleeping, LMIC, Bedtime, Waketime

## Abstract

**Objective:**

Sleep is an important marker of healthy development and has been associated with emotional, behavioral, and cognitive development. There is limited longitudinal data on children's sleep with only a few reports from low- and middle-income countries (LMICs). We investigate sleep parameters and associated sociodemographic characteristics in a population-based longitudinal study in Pelotas, Brazil.

**Methods:**

Data from the Pelotas 2004 Birth Cohort were used (*N* = 3842). Infant sleep was collected through maternal report at 3, 12, 24, and 48 months: sleep duration, bed and wake time, nighttime awakenings, co-sleeping and sleep disturbances (24 and 48 months).

**Results:**

Compared to children in high-income countries (HICs), children in Brazil showed a substantial shift in rhythms with later bed and wake times by approximately 2 hours. These remain stable throughout the first 4 years of life. This population also shows high levels of co-sleeping which remain stable throughout (49.0–52.2%). Later bedtime was associated with higher maternal education and family income. Higher rates of co-sleeping were seen in families with lower income and maternal education and for children who were breastfed. All other sleep parameters were broadly similar to data previously reported from HICs.

**Conclusion:**

The shift in biological rhythms in this representative community sample of children in Brazil challenges our understanding of optimal sleep routine and recommendations.

## Highlights

• Biological rhythms are shifted with later bedtime by approximately 2 h (22.18 hours).
• Later bedtime is associated with higher maternal education and family income.
• High levels of co-sleeping are prevalent until 4 years of age (49% at 3 months to 52% at 4 years).
• Co-sleeping was more prevalent in families with lower income and maternal education.

## 1 Introduction

Sleep is an important indicator of healthy development and one of the earliest markers of bio-behavioral organization. Sleep in young children, specifically sleep problems, has been associated both with current and future symptoms of emotional and behavioral problems, as well as cognitive development [1], [2], [3]. Higher levels of motor activity during sleep and more fragmented sleep (measured using actigraphy) have also been negatively associated with cognitive and language scores as early as 10 months of age using the Bayley Scales of Infant Development [4] as well as attention at 3–4 years of age [5]. Longitudinally, sleep problems have been associated with anxiety [6], aggression, social and attention problems. Better sleep consolidation and sleep duration are associated with higher language achievement [7], cognition function (executive function, school performance, and multiple domain cognitive functioning), better future performance on executive function tasks [8], fewer current internalizing, and externalizing symptoms [9].

Epidemiological studies have primarily reported data on sleep duration and awakenings in children but fewer studies have reported population level estimates for other sleep parameters. The limited longitudinal data available have almost exclusively stemmed from high-income countries (HICs) mostly Europe and the USA [13], [14], [15], [16]. Consequently, sleep recommendations have largely been proposed in HICs, but are still lacking a large empirical base [17]. In identifying normative patterns of sleep in children and in proposing sleep recommendations which may relate to sleep duration or hygiene, data from low- and middle-income country (LMIC) cohorts have an important part to play due to the specific cultural and socioeconomic factors that influence sleep and parental perceptions of sleep.

Sleep patterns result from the interaction of environmental and biological aspects that govern the sleep–wake rhythm. Identifying environmental factors associated with sleep parameters is important for two reasons: first, they can help identify individuals who may be at increased risk of disturbed sleep and second, they can be useful in assessing the likely effectiveness of interventions in specific groups or settings. Comparing sociodemographic factors associated with sleep parameters in different cohorts affords greater confidence in the strength of these associations but also potentially in identifying any causal link. Notably, the opportunity to compare these associations across different cohorts, especially of those from HICs vs LMICs where there may be different confounding structures gives further insight with regard to the generalizability of sleep norms and reference values across countries [18].

### 1.1 Aim

The aim of this paper is to present data from a large prospective ongoing birth cohort in Brazil of children born in 2004, to report population-based sleep parameters from infancy to four years of life and identify associated sociodemographic factors. We examine a range of sleep parameters to allow for comparability to previously published research (eg sleep duration and bed/wake times), to present normative patterns on parameters known to be predictive of later child development (eg sleep duration, awakenings, sleep disturbances) and to present normative data on culture-specific factors (co-sleeping) which are less common in HICs.

## 2 Materials and methods

### 2.1 Participants

This study used data from the Pelotas 2004 Birth Cohort. The study included all live births which took place from 1 January 2004 to 31 December 2004 in the city of Pelotas, Brazil. Births were identified through daily visits to the city's five maternity hospitals. Mothers were interviewed soon after delivery providing detailed information on demographic, socioeconomic, and behavioral characteristics. The study complies with ethical standards and principles of the Declaration of Helsinki and was approved by the Medical Ethics Committee of the Federal University of Pelotas. All women provided written informed consent at each follow-up. The study recruited 4231 participants at birth (<1% refusals). Data from four follow-ups are used for this analysis at 3, 12, 24, and 48 months. Sleep measures at the three-month follow-up were only collected for a randomly selected sub-sample of the cohort (*N* = 903). These mothers did not differ from the larger sample in any demographic characteristics. Overall, the follow-up rates ranged from 96% at age three months to 90% at age 48 months. Information was collected at home using paper questionnaires and included socioeconomic and demographic characteristics, feeding practices and child growth, maternal health, and child development. Interviewers read out loud the questions to the mother or caregiver. A detailed description of the sample can be found in Santos et al. [19].

### 2.2 Measures

#### 2.2.1 Sleep variables

Maternal reports of infant sleep were collected at 3, 12, 24, and 48 months. Seven variables of sleep behavior are described. Parents were asked to report on children's sleep in the past two weeks. Parents were asked for the bed and wake time (hh.min, “In the last two weeks, what time did the child go to bed?” and “What time did the child wake up?”). These were used to calculate nighttime sleep duration. Parents were also asked “In the last two weeks, more or less how many times did the child sleep during the day?” and “How long, more or less did the child sleep each time”. These two variables were used to calculate daytime sleep duration. Sleep duration over the 24-h period (total sleep duration) was calculated by adding the nighttime and daytime sleep duration variables. Parents were asked whether “in the last two weeks the child woke up in the night?”, “How many nights in the last two weeks?”, and “How often per night?” (nighttime awakenings). Parents were also asked to report whether the child sleeps in the same bed with someone else (co-sleeping). Sleep disturbances were calculated for 24 and 48 months and were intended to indicate behaviors which may increase the risk of future sleep problems. We used the following variables as indicators of potential sleep disturbances (sum of yes/no answers): the child has nightmares/night terrors, the child has restless sleep, child experiences difficulty going to sleep, child wakes up at night, and child woke up early (bottom 10% of early wakers) (potential total score of 5). This scale was derived from variables which are good indicators of potential sleep problems and is modeled on a sleep problems scale used in the ALSPAC cohort study [20]. When parents were asked about potential sleep problems there was no specified time period.

#### 2.2.2 Clinical and demographic variables

The following variables were included in this analysis:

Maternal Factors: maternal skin color (white, black, other), age at delivery (≤19 years, 20–34 years, and ≥35 years), prenatal smoking (no/yes), prenatal alcohol (no/yes), maternal education (0–4 years, 5–8 years, ≥9 years), family income (categorized in quintiles), and parity (1, 2, 3, or more).
Child Factors: sex (male/female), gestational age at birth (≤28 weeks, 28–32 weeks, 32–36 weeks, and ≥37 weeks), birth weight in grams (<2500 g, ≥2500 g), and neonatal complications (APGAR scores at 5 min <7).
Environmental/Other Factors: type of delivery (vaginal, cesarean, collected at birth), does the child sleep alone (no/yes, collected at each time point), breastfeeding (no/yes, collected at 3, 12, and 24 months), time spent watching TV (total in the day), and time spent watching TV at nighttime (collected at 24 and 48 months of age). The top 15% of values were categorized as watching more TV. For TV at nighttime this was 90 or more minutes at 24 months and 120 or more minutes at 48 months. For total time watching TV in the day this was 210 or more minutes at 24 months and 350 or more minutes at 48 months. We also included data on whether there were other children living in the home (siblings or other) who were younger or older than the study child (entered in the model as yes/no for children under the age of the study child and yes/no for children older than the study child) at 24 and 48 months of age.

### 2.3 Statistical analysis

First, we examined the descriptives for each sleep variable. We modeled change in sleep across time using growth curve models (STATA command xtmixed). Associations between sleep variables (outcome) and sociodemographic characteristics were examined using hierarchical linear (for sleep duration variables and bed/wake times) and Poisson regressions (for co-sleeping, awakenings, sleep disturbances). The multivariable analysis was determined a priori and the sociodemographic variables were entered in three steps: (1) maternal characteristics, (2) child characteristics, and (3) environmental/other factors (in the order presented in the section above). For awakenings at 24 and 48 months, the earlier awakenings (at 12 and 24 months) were also added to the model (Step 4). The variables included were decided on a conceptual framework. To correct for the effect of multiple tests a significance of *p* ≤ 0.01 was adopted. All analysis was performed using STATA 13.

## 3 Results

### 3.1 Attrition analysis

We examined differences in sociodemographic factors for participants with sleep data available (for at least one sleep variable, *N* = 3842) compared to participants with no sleep data available (*N* = 309). We used the 12-month data for comparison because the 3-month data was only collected for a sub-sample of the cohort (Supplementary Table S1, *N* = 3842). Comparison of sociodemographic characteristics revealed differences in family income and maternal years of schooling, with a higher proportion of women in the lower-income quintiles (lower two income quintiles 52.8%) and of women with 0–4 years of schooling (19.4%) in the group of women for whom no data was available at 12 months compared to women with infant sleep data available (lower two income quintiles 39.8% and 0–4 years of schooling 15.1%). Furthermore, women who had dropped out were more likely to smoke and drink alcohol during pregnancy. Finally, there was a higher proportion of children born before 37 weeks of gestation in the group with no sleep data available compared to children with sleep data available (23.7% vs 36.2%).

### 3.2 Sleep duration

Table 1 presents the descriptives for sleep variables and Table 2 the results from the growth curve modeling and pairwise comparisons across time. Total sleep duration (in the 24-h period) decreased significantly at each time point from 13.34 h (95% CI 13.22, 13.42) to 11.01 h (95% CI 10.96, 11.07) at 48 months (Table 1). Nighttime sleep duration showed an overall significant increase from 3 to 48 months (3-month mean = 9.38 h, 95% CI 9.29, 9.46; 48-month mean = 10.37 h 95% CI 10.33, 10.41), as is expected developmentally with nighttime sleep consolidation (Table 1). Day Time sleep duration, as expected, showed significant declines at each time point. At 3 months 99.34% of the sample reported daytime sleeping (mean = 3.84 h 95% CI 3.76, 3.92) and by 48 months this had decreased to 40.22% of the sample reporting daytime napping (mean = 0.62 h, 95% CI 0.58, 0.66).

**Table 1:** Descriptive analysis for bedtime and waketime (presented as clock time) and nighttime awakenings, sleep duration variables (presented in decimals).

Age	*N* (%)	Mean	SD	Min–Max	Median	2.5%	33%	66%	97.5%	
**Bedtime (in decimals)**										
3 months	866 (95.9)	22.26	2.63	18.00–02.00	22.00	20.00	21.50	23.00	1.00	
12 months	3810 (90.0)	22.11	1.23	18.15–02.00	22.00	20.00	21.50	22.50	1.00	
24 months	3792 (89.6)	22.19	1.08	19.00–02.00	22.00	20.00	21.50	22.50	0.00	
48 months	3719 (87.9)	22.17	1.22	18.30–02.00	22.00	19.75	21.50	22.50	1.00	
**Waketime (in decimals)**										
3 months	866 (95.9)	7.65	0.35	05.00–11.30	7.50	6.00	7.00	8.00	10.50	
12 months	3810 (90.0)	8.21	0.37	04.00–12.00	8.00	6.00	7.50	9.00	11.00	
24 months	3792 (89.6)	8.54	0.32	05.00–12.00	8.50	6.00	8.00	9.00	11.00	
48 months	3719 (87.9)	8.53	0.37	04.30–12.30	8.52	6.00	7.75	9.00	11.00	
**Awakenings (mean number of episodes per night)**										
3 months	866 (95.9)	1.32	1.04	0.00–4.00	1	0	1	2	4	
12 months	3806 (90.0)	1.24	1.29	0.00–6.00	1	0	0	2	4	
24 months	3769 (89.1)	0.77	1.01	0.00–5.00	0	0	0	1	3	
48 months	3724 (88.0)	0.62	0.82	0.00–5.00	0	0	0	1	3	
**Total sleep duration**										
3 months	844 (93.5)	13.33	2.91	5.50–22.00	13	8.35	12	14.13	19.50	
12 months	3772 (89.2)	12.29	1.81	6.75–18.00	12.25	8.97	11.50	13	16	
24 months	3565 (84.3)	11.96	1.36	8.00–16.00	12	9	11.50	12.50	14.75	
48 months	3651 (86.3)	11.02	1.28	7.00–15.00	11	8.50	10.50	11.50	14	
**Sleep duration – nighttime**										
3 months	844 (93.5)	9.38	1.69	4.50–14.00	9.5	6	8.50	10	12.78	
12 months	3772 (89.2)	10.10	1.33	6.00–14.00	10	7.48	9.50	10.50	12.75	
24 months	3565 (84.3)	10.37	1.19	6.75–14.00	10.5	8	10	11	12.50	
48 months	3651 (86.3)	10.37	1.21	6.67–14.00	10.5	8	10	11	12.50	
Age	*N* (%)	Mean^a^	SD^a^	Min–Max	Median	2.50%	33%	66%	97.50%	Daytime napping %^b^
**Daytime sleep duration (including those who did not sleep in the day)**										
3 months	844 (93.5)	3.84	2.42	0.00–10.00	3	0.50	2	4.50	9	99.34%
12 months	3772 (89.2)	2.17	1.35	0.00–6.00	2	0.33	1.50	2.25	6	98.97%
24 months	3565 (84.3)	1.57	0.91	0.00–4.00	1.50	0	1	2	3	90.70%
48 months	3651 (86.3)	0.62	0.88	0.00–3.50	0	0	0	1	3	40.22%

SD, standard deviation.

a Using the whole sample (children who slept and those who did not sleep during the day).

b The percentage of children who slept during the day.

**Table 2:** Growth curve modeling of sleep variables and pairwise comparisons.

Sleep variable	Time points	Margins	95% CI	Between subject mean change at the different time points compared to reference			Pairwise comparisons^a^			
				Coefficient	95% CI			Coefficient	95% CI	
**Bedtime**										
	3 months *(intercept)*	22.26**	22.19–22.34	REF						
	12 months	22.11	22.07–22.15	−0.15**	−0.23	−0.08				
	24 months	22.19	22.15–22.23	−0.07	−0.15	0.00	24 months vs 12 months	0.08**	0.04	0.13
	48 months	22.17	22.13–22.21	−0.09	−0.17	−0.02	48 months vs 24 months	−0.02	−0.06	0.02
**Waketime**										
	3 months *(intercept)*	7.65**	7.57–7.73	REF						
	12 months	8.21	8.17–8.25	0.56**	0.47	0.64				
	24 months	8.54	8.49–8.58	0.88**	0.8	0.97	24 months vs 12 months	0.33**	0.28	0.38
	48 months	8.53	8.49–8.58	0.88**	0.8	0.96	48 months vs 24 months	−0.01	−0.05	0.04
**Awakenings**										
	3 months *(intercept)*	1.32**	1.25–1.39	*REF*						
	12 months	1.24	1.21–1.28	−0.07	−0.15	0.00				
	24 months	0.77	0.74–0.81	−0.54**	−0.62	−0.47	24 months vs 12 months	−0.47**	−0.05	−0.04
	48 months	0.62	0.58–0.65	−0.7**	−0.78	−0.63	48 months vs 24 months	−0.016**	−0.20	−0.11
**Total sleep**										
	3 months *(intercept)*	13.33**	13.22–13.44	REF						
	12 months	12.29	12.24–12.34	−1.04**	−1.15	−0.92				
	24 months	11.96	11.91–12.01	−1.37**	−1.49	−1.25	24 months vs 12 months	−0.33**	−0.40	−0.26
	48 months	11.02	10.96–11.07	−2.31**	−2.43	−2.20	48 months vs 24 months	−0.94**	−1.01	−0.87
**Night sleep**										
	3 months *(intercept)*	9.38**	9.29–9.46	REF						
	12 months	10.10	10.06–10.14	0.73**	0.64	0.82				
	24 months	10.37	10.33–10.41	1.00**	0.91	1.09	24 months vs 12 months	0.27**	0.22	0.32
	48 months	10.37	10.33–10.41	0.99**	0.9	1.08	48 months vs 24 months	0.00	−0.06	0.05
**Day sleep**										
	3 months *(intercept)*	3.84**	3.76–3.92	REF						
	12 months	2.17	2.14–2.21	−1.66**	−1.75	−1.57				
	24 months	1.57	1.53–1.60	−2.27**	−2.36	−2.18	24 months vs 12 months	−0.61**	−0.66	−0.56
	48 months	0.62	0.58–0.66	−3.21**	−3.3	−3.13	48 months vs 24 months	−0.94**	−0.99	−0.89
**Sleep disturbances**										
	24 months	1.30**	1.27–1.34	REF						
	48 months	1.24	1.21–1.28	−0.06*	−0.1	−0.02				

CI, confidence interval.

*p < 0.01, **p < 0.001.

a Pairwise comparisons for 12 months vs 3 months appear in the first column where 3 months is the reference group.

There was small variation across time points in Bedtime (3 months, 22.26; and 48 months, 22.17) (Table 2). Bedtime showed a small yet significant decrease from 3 to 12 months (−0.15, 95% CI −0.23, −0.08 min) and an increase from 12 to 24 months (0.08, 95% CI 0.04, 0.13 min) but no change between 24 and 48 months. There was an overall significant increase in waketime (three months, 7.63, and 48 months, 8.53). Waketime increased from 3 to 12 months (0.56 h 95% CI 0.47, 0.64) and from 12 to 24 months (0.33, 95% CI 0.28, 0.38). There was no difference between waketime at 24 and 48 months (Table 2).

### 3.3 Awakenings

**Fig. 1:**
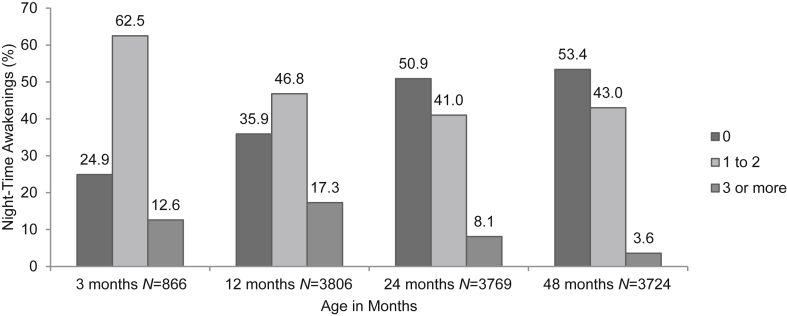
Awakenings (shown as %) at four time points in childhood.

The mean number of awakenings decreased significantly at each time point from 1.32 (95% CI 1.25, 1.39) at three months to 0.62 (95% CI 0.58, 0.65) at 48 months (Table 1, Table 2), and by 48 months of age 53.4% of the sample were reporting zero awakenings. In Fig. 1, we present pictorially the percentage of children with zero awakenings, one to two awakenings, and three or more awakenings. Only 3.6% of the sample reported waking up three or more times by 48 months of age. Focusing on children who displayed three or more awakenings at 24 months (8.1%), by 48 months 42.9% of them woke up once or twice and 3.6% of them continued to experience three or more awakenings per night (data not shown).

### 3.4 Sleep disturbances

Sleep disturbances showed a small yet significant decrease from 24 to 48 months of age (mean at 24 months 1.3, 95% CI 1.27, 1.34, change from 24 to 48 months, −0.06 95% CI −0.1, −0.02) (Table 2). Nearly half the children had no sleep disturbances at 24 (42.3%) and 48 months (44.7%) (Table 3). Only a small percentage of children experienced three or more disturbances; this decreased from 6.8% at 24 months to 5% at 48 months (*p* = 0.0006). Awakenings at night and restless sleep were the most frequently reported sleep disturbances.

**Table 3:** Descriptive analysis of sleep disturbances.

	24 months	48 months
	*N* (%)	*N* (%)
Sleep disturbances		
0	1618 (42.3)	1690 (44.7)
1	1318 (34.4)	1295 (34.3)
2	628 (16.4)	606 (16.0)
3	219 (5.7)	169 (4.5)
4	44 (1.1)	20 (0.5)
5	1 (0.0)	
Total	3828 (100.0)	3780 (100.0)
Nightmares (yes)	522 (13.49)	527 (13.87)
Is child sleep restless (yes)	1366 (35.3)	1326 (34.9)
Difficulty going to sleep (yes)	1315 (33.66)	795 (20.92)
Child wakes up at night (yes)	1842 (48.9)	1727 (46.4)
Child woke up early (yes)	328 (8.6)	324 (8.7)

### 3.5 Demographic characteristics associated with sleep variables

#### 3.5.1 Sleep duration

Overall there were no consistent associations between sleep duration and sociodemographic characteristics. Total sleep duration at 24 months was associated with having other children in the home and at 48 months was negatively associated with time spent watching TV at nighttime (B = −0.06, 95% CI −0.09, −0.03) (Supplementary Table S2). There was weak evidence that sleep duration during the day (Supplementary Table S3) was associated with child gender at 24 months (B = −0.06, 95% CI −0.09, −0.02) with boys sleeping more during the day. At 48 months, sleep during the day was also associated with co-sleeping and family income; children who co-slept and children from families with lower income slept longer during the day. Night-time sleep duration was associated with maternal age; children of younger mothers (≤19 years) slept longer compared to children of older mothers (≥35 years) at 12 and 24 months (B = 0.11, 95% CI 0.04, 0.18; and B = 0.10, 95% CI 0.03, 0.17, respectively) (Supplementary Table S4). Finally, at 48 months watching ≥120 min of TV at nighttime was associated with shorter sleep duration (B = −0.14, 95% CI −0.18, −0.09) and having younger children at home was associated with longer sleep duration (B = 0.06, 95% CI 0.02, 0.1).

There was a consistent and positive association between both bedtime and waketime, and maternal schooling years, suggesting that children of more educated mothers went to bed and woke up later compared to women with 0–4 years of schooling (range of B −0.13, −0.17) (Table 4, Table 5). Family income was also significantly associated with bed and wake times at 12, 24, and 48 months (range of B −0.09, −0.14) with children in families with higher income going to bed and waking up later compared to children in the lower two income quintiles.

**Table 4:** Hierarchical linear regression analysis for bedtime and sociodemographic characteristics from 3 to 48 months.

	3 months *N* = 843			12 months *N* = 3600			24 months *N* = 3057			48 months *N* = 3446		
	B	95% CI		B	95% CI		B	95% CI		B	95% CI	
		Lower	Upper		Lower	Upper		Lower	Upper		Lower	Upper
**Maternal characteristics**												
*Maternal skin color*												
White	−0.08	−0.25	0.08	−0.07	−0.14	0.01	0.02	−0.05	0.09	−0.03	−0.1	0.05
Black	−0.06	−0.22	0.1	0.01	−0.07	0.08	0.04	−0.03	0.11	−0.01	−0.08	0.07
Other	REF			REF			REF			REF		
*Maternal age (years)*												
≤19	0.1	−0.05	0.26	0.03	−0.04	0.1	0.02	−0.05	0.09	0.02	−0.04	0.09
20–34	0.01	−0.13	0.16	−0.01	−0.08	0.05	0	−0.06	0.06	−0.01	−0.07	0.05
≥35	REF			REF			REF			REF		
*Smoked during pregnancy (no)*	0	−0.09	0.1	−0.04	−0.08	0	−0.01	−0.05	0.04	−0.02	−0.06	0.02
*Alcohol during pregnancy (no)*	0.06	−0.03	0.14	−0.02	−0.06	0.03	0	−0.04	0.04	0	−0.04	0.04
*Maternal schooling (years)*												
0–4	−0.19*	−0.32	−0.07	−0.13**	−0.19	−0.08	−0.14**	−0.2	−0.09	−0.13**	−0.18	−0.08
5–8	−0.06	−0.18	0.05	−0.03	−0.08	0.02	−0.07	−0.12	−0.02	−0.03	−0.08	0.02
≥9	REF			REF			REF			REF		
*Parity*												
1	0.01	−0.12	0.15	0.07	0.01	0.13	0.01	−0.06	0.08	0.04	−0.02	0.11
2	0.07	−0.05	0.18	0.02	−0.03	0.07	0.02	−0.03	0.07	0.03	−0.02	0.08
≥3	REF			REF			REF			REF		
*Family income quintiles*												
1	−0.09	−0.22	0.04	−0.13**	−0.19	−0.06	−0.13**	−0.19	−0.07	−0.14**	−0.2	−0.08
2	−0.06	−0.18	0.07	−0.09*	−0.15	−0.03	−0.11**	−0.17	−0.05	−0.06	−0.12	0
3	−0.04	−0.16	0.09	−0.07	−0.13	−0.01	−0.07	−0.12	−0.01	−0.03	−0.08	0.03
4	0	−0.12	0.11	−0.05	−0.11	0.01	−0.03	−0.09	0.02	0	−0.05	0.05
5	REF			REF			REF			REF		
**Child characteristics**												
*Child gender (male)*	0.07	−0.02	0.16	0.05	0.00	0.09	0.03	−0.01	0.08	0.02	−0.02	0.06
*Gestational age at birth (weeks)*												
≤28	0.08	−0.09	0.24	−0.02	−0.1	0.06	−0.09	−0.17	−0.02	0.07	−0.01	0.15
29–32	0	−0.09	0.09	0.02	−0.03	0.07	−0.03	−0.08	0.02	0.01	−0.04	0.06
33–36	0.05	−0.05	0.15	−0.05	−0.1	−0.01	−0.02	−0.06	0.02	0.03	−0.02	0.07
≥37	REF			REF			REF			REF		
*Birth weight (g) < 2500*	0.02	−0.1	0.13	0.03	−0.02	0.08	0.01	−0.04	0.06	0.02	−0.03	0.07
*Neonatal complications (APGAR < 7)*	−0.02	−0.11	0.07	−0.01	−0.05	0.04	−0.06	−0.11	−0.01	0.02	−0.03	0.06
**Environmental/other factors**												
*Mode of delivery (normal)*	−0.01	−0.1	0.09	−0.02	−0.06	0.03	−0.04	−0.09	0	−0.06*	−0.11	−0.02
*Co-sleeping (yes)*	0.04	−0.06	0.14	−0.04	−0.09	0	−0.05	−0.1	0.01	−0.04	−0.08	−0.00
*Breastfeeding (no)*	−0.09	−0.18	0.01	0	−0.11	0.11	−0.04	−0.13	0.05			
*Breastfeeding (yes)*	REF			0.05	−0.06	0.17	−0.07	−0.16	0.02			
*Never breastfed*				REF			REF					
*TV watching total*							0.02	−0.03	0.07	0.06*	0.02	0.1
*TV at nighttime*							0.05	0.01	0.10	0.22**	0.18	0.27
*Younger children at home (yes)*							−0.09**	−0.13	−0.04	−0.12**	−0.16	−0.08
*Older children at home (yes)*							−0.06	−0.12	−0.01	−0.07	−0.12	−0.02
	R^2^ = 0.05, F(23,8199) = 1.76			R^2^ = 0.03, F(24,3575) = 5.24			R^2^ = 0.06, F(28,3028) = 6.96			R^2^ = 0.09, F(26,3419) = 13.49		

CI, confidence interval.

*p < 0.01, **p ≤ 0.001.

**Table 5:** Hierarchical linear regression analysis for waketime and sociodemographic characteristics from 3 to 48 months.

	3 months *N* = 842			12 months *N* = 3614			24 months *N* = 3066			48 months *N* = 3445		
	B	95% CI		B	95% CI		B	95% CI		B	95% CI	
		Lower	Upper		Lower	Upper		Lower	Upper		Lower	Upper
**Maternal characteristics**												
*Maternal skin color*												
White	−0.01	−0.18	0.17	−0.05	−0.13	0.03	0.01	−0.07	0.09	0.01	−0.07	0.09
Black	0.01	−0.16	0.18	−0.01	−0.09	0.07	0	−0.08	0.08	−0.04	−0.12	0.04
Other	REF			REF			REF			REF		
*Maternal age (years)*												
≤19	0.15	−0.01	0.31	0.14**	0.07	0.21	0.12*	0.04	0.2	0.02	−0.06	0.09
20–34	0.05	−0.1	0.2	0.06	−0.01	0.12	0.03	−0.03	0.1	0.01	−0.06	0.08
≥35	REF			REF			REF			REF		
*Smoked during pregnancy (no)*	−0.03	−0.13	0.07	−0.03	−0.08	0.01	−0.01	−0.05	0.04	−0.01	−0.06	0.03
*Alcohol during pregnancy (no)*	0	−0.09	0.09	0.02	−0.02	0.07	−0.03	−0.08	0.02	0	−0.04	0.05
*Maternal schooling (years)*												
0–4	−0.16	−0.28	−0.03	−0.17**	−0.22	−0.11	−0.17**	−0.23	−0.11	−0.14**	−0.19	−0.08
5–8	−0.13	−0.25	−0.01	−0.07	−0.12	−0.01	−0.05	−0.1	0.01	−0.01	−0.07	0.04
≥9	REF			REF			REF			REF		
*Parity*												
1	−0.02	−0.17	0.12	0.08*	0.02	0.15	−0.05	−0.13	0.03	0.03	−0.04	0.1
2	−0.03	−0.15	0.09	0.04	−0.01	0.09	−0.02	−0.07	0.04	0.02	−0.03	0.08
≥3	REF			REF			REF			REF		
*Family income quintiles*												
1	0.04	−0.09	0.18	−0.09*	−0.15	−0.02	−0.1*	−0.17	−0.04	−0.1*	−0.17	−0.04
2	−0.08	−0.21	0.05	−0.09*	−0.15	−0.02	−0.08	−0.14	−0.01	−0.09*	−0.15	−0.02
3	0.02	−0.11	0.15	−0.03	−0.09	0.03	−0.04	−0.1	0.02	−0.03	−0.09	0.03
4	0.08	−0.04	0.19	0	−0.06	0.06	−0.01	−0.07	0.05	0	−0.06	0.06
5	REF			REF			REF			REF		
**Child characteristics**												
*Child gender (male)*	0.11	0.02	0.21	0.08**	0.04	0.12	0.06*	0.02	0.11	0.1**	0.06	0.15
*Gestational age at birth (weeks)*												
≤28	−0.12	−0.29	0.05	0	−0.08	0.08	0.01	−0.07	0.1	0.09	0	0.17
29–32	−0.02	−0.11	0.08	−0.02	−0.07	0.03	0	−0.05	0.06	0.00	−0.05	0.05
33–36	−0.1	−0.21	0	−0.06*	−0.11	−0.02	−0.01	−0.06	0.04	0.03	−0.02	0.07
≥37	REF			REF			REF			REF		
*Birth weight (g) < 2500*	0.05	−0.07	0.17	−0.03	−0.08	0.03	−0.01	−0.06	0.05	0.00	−0.06	0.05
*Neonatal complications (APGAR < 7)*	−0.01	−0.11	0.09	0.01	−0.04	0.06	−0.02	−0.07	0.03	0.02	−0.03	0.07
**Environmental/other factors**												
*Mode of delivery (normal)*	−0.02	−0.12	0.08	−0.02	−0.06	0.03	−0.1**	−0.15	−0.05	−0.03	−0.07	0.02
*Co-sleeping (yes)*	−0.14	−0.24	−0.03	0.04	−0.01	0.09	−0.03	−0.08	0.02	−0.05	−0.10	−0.00
*Breastfeeding (no)*	0.01	−0.09	0.11	0.02	−0.09	0.14	−0.04	−0.14	0.06			
*Breastfeeding (yes)*	REF			0.08	−0.04	0.19	0.00	−0.1	0.1			
*Never breastfed*				REF			REF					
*TV watching total*							0.02	−0.03	0.08	0.07*	0.02	0.12
*TV at nighttime*							0.06	0.01	0.11	0.08**	0.03	0.13
*Younger children at home (yes)*							0.01	−0.04	0.05	−0.05	−0.1	−0.01
*Older children at home (yes)*							−0.11**	−0.17	−0.04	−0.04	−0.09	0.02
	R^2^ = 0.05, F(23,818) = 1.67			R^2^ = 0.05, F(24,3589) = 7.18			R^2^ = 0.05, F(28,3037) = 5.92			R^2^ = 0.05, F(26,3418) = 6.73		

CI, confidence interval.

*p < 0.01, **p ≤ 0.001.

TV watching at nighttime was associated with later bedtime at 24 and 48 months (B = 0.07, 95% CI 0.01, 0.13; B = 0.23, 95% CI 0.19, 0.28) and later waketime at 48 months. Child gender at 12, 24, and 48 months was associated with waketime (B range 0.06, 0.10) with boys waking up earlier than girls. Additionally, we found evidence of an association between waketime and maternal age at 12 and 24 months with later wake times for children of younger mothers (≤19 years) compared to older mothers (≥35 years). Having other children in the home was also associated with bed and wake times: having children younger than the study child in the home was associated with earlier bed times at 24 and 48 months (B range −0.09, −0.12) and having children older than the study child in the home was associated with earlier waketime at 24 months (B = −0.11, 95% CI −0.17, −0.04).

There was a persistent association between co-sleeping and maternal skin color through all time points with slightly increased rates of co-sleeping in non-white mothers (Supplementary Table S5). There was also a persistent association between co-sleeping and family income with significantly higher prevalence rates of co-sleeping in families in the lower income quintiles (for example, at 24 months the prevalence ratio at quintile 1 was 39% (95% CI 15, 69%) higher and at 48 months the prevalence ratio at quintile 1 was 52% (95% CI 25, 84%) higher compared to the highest-income quintile).

At 12 and 24 months, co-sleeping was also associated with maternal age where co-sleeping was more prevalent in the younger groups (prevalence in mothers ≤19 years old was 50% higher (95% CI 20, 87%) higher, and in 20- to 34-year-olds, 25% (95% CI 5, 48%) compared to women 35 years or older). At these time points co-sleeping was also higher in children who were breastfed (at 24 months the prevalence was 35% higher than among those that were not breastfed). Maternal schooling was also associated with co-sleeping in the first year of life (at 12 months, prevalence among infants of mothers who had 0–4 years of education was 34% higher compared to those of mothers with nine years or more of education).

#### 3.5.2 Awakenings

At three months of age, boys were 27% more likely to wake at night (95% CI 5, 41%) compared to girls. At 12 and 24 months of age, awakenings were associated with breastfeeding, with a higher incidence ratio of awakenings in children who were being breastfed (1.45–1.74) compared to those never breastfed. Awakenings at 12 months were associated with maternal alcohol consumption during pregnancy, with children of mothers who did not consume alcohol during pregnancy being 23% less likely to experience awakenings (95% CI 1.10, 1.33). Children with one or more awakenings at 12 or 24 months were more likely to also wake up during the night at 24 or 48 months, respectively, compared to children who had no awakenings; for example, children with three awakenings at 24 months were 66% more likely to wake up during the night at 48 months (95% CI 1.38, 2.3) (Table 6).

**Table 6:** Poisson regression to examine the associations between awakenings and sociodemographic characteristics.

	3 months *N* = 802			12 months *N* = 3582			24 months *N* = 2797			48 months *N* = 2812		
	B	95% CI		B	95% CI		B	95% CI		B	95% CI	
		Lower	Upper		Lower	Upper		Lower	Upper		Lower	Upper
**Maternal characteristics**												
*Maternal skin color*												
White	0.94	0.73	1.20	1.06	0.94	1.19	0.98	0.82	1.17	1.04	0.85	1.26
Black	0.99	0.76	1.29	0.97	0.85	1.10	1.11	0.91	1.35	1.04	0.84	1.29
Other	1^a^			1^a^			1^a^			1^a^		
*Maternal age (years)*												
≤19	0.84	0.64	1.11	1.07	0.94	1.21	0.80	0.67	0.97	0.95	0.79	1.15
20–34	1.05	0.85	1.29	1.10	1.01	1.21	0.95	0.83	1.09	1.04	0.93	1.20
≥35	1^a^			1^a^			1^a^			1^a^		
*Smoked during pregnancy*												
No	1.04	0.90	1.20	1.02	0.95	1.09	0.96	0.86	1.06	1.04	0.94	1.16
Yes	1^a^			1^a^			1^a^			1^a^		
*Alcohol during Pregnancy*												
No	0.72	0.55	0.96	0.77**	0.66	0.90	1.27	0.94	1.71	1.03	0.76	1.16
Yes	1^a^			1^a^			1^a^			1^a^		
*Maternal schooling (years)*												
0–4	1.15	0.92	1.42	1.10	0.99	1.21	1.25**	1.07	1.45	0.97	0.82	1.14
5–8	0.94	0.80	1.10	1.05	0.98	1.13	1.10	0.98	1.24	1.00	0.90	1.12
≥9	1^a^			1.00			1^a^			1^a^		
*Parity*												
1	1.22	1.01	1.48	0.93	0.85	1.01	1.20	1.03	1.41	1.08	0.93	1.25
2	1.12	0.94	1.34	1.01	0.93	1.09	1.06	0.94	1.21	0.94	0.83	1.06
≥3	1^a^			1.00			1^a^			1^a^		
*Family income quintiles*												
1	1.16	0.94	1.45	1.06	0.96	1.18	0.96	0.81	1.13	0.84	0.71	0.99
2	1.07	0.87	1.32	1.01	0.91	1.12	0.90	0.77	1.06	0.84	0.72	0.98
3	1.08	0.87	1.33	0.94	0.85	1.05	0.89	0.76	1.04	0.94	0.82	1.09
4	0.98	0.80	1.19	1.02	0.93	1.13	0.92	0.79	1.06	0.83	0.73	0.95
5	1^a^			1^a^			1^a^			1^a^		
**Child characteristics**												
*Child gender*												
Male	1.27**	1.12	1.44	1.07	1.01	1.13	0.92	0.84	1.01	1.02	0.93	1.11
Female	1^a^			1^a^			1^a^			1^a^		
*Gestational age at birth (weeks)*												
≤28	2.19	0.92	5.23	1.74	1.01	2.99	1.99	1.02	3.88	0.97	0.41	2.32
29–32	1.08	0.69	1.68	1.17	0.90	1.51	0.98	0.53	1.82	0.77	0.51	1.16
33–36	1.02	0.87	1.20	1.05	0.97	1.13	1.11	1.00	1.24	1.02	0.92	1.14
≥37	1^a^			1^a^			1^a^			1^a^		
*Birth weight (g)*												
<2500	1.11	0.87	1.43	0.93	0.82	1.05	0.83	0.68	1.02	1.13	0.98	1.38
≥2500	1^a^			1^a^			1^a^			1^a^		
*Neonatal complications (APGAR score)*												
<7	1.08	0.71	1.67	1.24	1.01	1.53	1.07	0.77	1.50	1.09	0.74	1.62
≥7	1^a^			1^a^			1^a^			1^a^		
**Environmental/other factors**												
*Mode of delivery*												
Normal	1.14	1.00	1.30	1^a^	0.94	1.07	1.04	0.5	1.15	0.89	0.82	0.98
Cesarean	1^a^			1^a^			1^a^			1^a^		
*Co-sleeping*												
No	0.94	0.82	1.07	1.03	0.97	1.10	0.96	0.87	1.06	0.85	0.77	0.94
Yes	1^a^			1^a^			1^a^			1^a^		
*Breastfeeding*												
No	1.07	0.93	1.23	1.07**	0.90	1.27	1.06**	0.82	1.37			
Yes	1^a^			1.74	1.46	2.07	1.45	1.11	1.90			
Never breastfed				1^a^			1^a^					
*TV watching total*												
Less							1.00	0.94	1.04	1.00	0.95	1.06
more							1^a^			1^a^		
*TV at nighttime*												
Less							0.98	0.93	1.04	1.02	0.97	1.07
more							1^a^			1^a^		
*Younger children at home*												
No							0.99	0.94	1.04	1.03	0.98	1.07
Yes							1			1		
*Older children at home*												
No							1.05	0.99	1.11	1.06	1.00	1.13
Yes							1			1		
*Awakenings at 12 months*												
0							1^a^			1^a^		
1							1.40	1.23	1.59	0.98	0.87	1.10
2							1.82	1.59	2.09	1.17	1.03	1.34
3							2.18	1.87	2.53	1.22	1.05	1.41
4							2.07	1.66	2.59	1.35	1.09	1.67
5							2.33	1.68	3.23	1.25	0.84	1.87
6							2.10	1.20	3.65	2.04	1.34	3.11
*Awakenings at 24 months*												
0										1^a^		
1										1.25	1.13	1.38
2										1.56	1.36	1.79
3										1.66	1.38	2.00
4										1.66	1.20	2.30
5										2.15	1.13	4.09

CI, confidence interval.

*p < 0.01, **p ≤ 0.001.

a Reference group.

#### 3.5.3 Sleep disturbances

At 48 months, children watching less TV at night (<120 min) were 12% (95% CI 5, 18%) less likely to experience sleep disturbances compared to those who watched 120 min or more per night (Supplementary Table S6). At 24 months, study children without older children in the home were 13% (95% CI 1.03, 1.23) more likely to experience sleep disturbances.

## 4 Discussion

This study describes normative sleep parameters and associated sociodemographic characteristics in a large prospective cohort in Brazil. Importantly, our research suggests sleep parameters in the cohort to be broadly similar to HICs in terms of means but are shifted on average 2 hours later with bedtime at approximately 22:18 throughout the first four years of life. We also report high and stable levels of co-sleeping across the first four years of life. This study offers important data on what constitutes ‘normal’ sleep and challenges our understanding of sleep recommendations and sleep hygiene, where earlier bedtimes are encouraged. Bedtime for all age groups was approximately 22:18 which is significantly later than bedtime reported in longitudinal cohorts in HICs (on average 20:00) [15], [21]. These bedtimes are not dissimilar to data from one multi-country study; in Hong Kong, Taiwan, Korea, and India bedtime was also on average around 22.00 [22] or to data from the Middle East [23]. We also found slightly later waketimes (on average 08:15) compared to UK (07:10) and Australia (07:15) as well as compared to waketimes reported in Hong Kong, Korea, and Taiwan (07:50) [22], but similar to those reported in a recent study in the Middle East [23]. The bed and wake times reported here for children are in line with data on adult samples in Brazil. It is therefore possible that infants are largely following the rhythms of their parents and families and that these result in different bed and wake times across countries and cultures [24]. This cohort also demonstrated high levels of co-sleeping with little change from 3 to 48 months of age (49.24–52.2%). These rates were higher compared to HICs which reported, for example, rates between 20% at 2 months of age and 10.5% at 24 months of age [3], [14] and are more similar to Asian or Middle Eastern countries (64.7% and 40.2%, respectively) [22], [23]. Awakenings are regularly considered an indicator of disturbed sleep. Awakenings at 24 months were associated with an increased risk of awakenings at 48 months; for example, children waking up three or more times at 24 months were 66% more likely to wake up during the night at 48 months. Reassuringly for many parents, awakenings declined over time as is expected developmentally, with a large proportion of children waking up more than three times at 24 months of age exhibiting one to two awakenings by 48 months (43%), and only a small portion of the sample displaying persistent awakenings (3.6%). In relation to other cohorts [3], [14], children in the Pelotas cohort overall showed a higher number of awakenings. For example, compared to a Dutch prospective cohort at 24 months, 6.3% of children woke up three or more times per night compared to 8.1% in the Pelotas cohort. Sleep disturbances were prevalent in this sample with 55.3% of the children scoring positively for at least one problem at 48 months compared to 88% of children in the ALSPAC cohort at 30 months of age [20]. Cultural issues are likely to be important in what parents perceive to be problematic sleep, and prior expectations relating to sleep behavior and hygiene are also likely to be key in assessing the prevalence of sleep disturbances across settings. A small number of studies have reported cross-cultural differences [25], [26] and further highlight the need for longitudinal data from LMICs. One study which compared sleep parameters across 23 countries from birth to 36 months of age reported significant cross-country differences [22]. Children from LMICs (predominantly Asian countries) had later bed and wake times, less nighttime sleep, less total sleep time, and higher rates of co-sleeping. Our analysis of sleep variables and associated sociodemographic characteristics revealed few consistent patterns across time points. Overall, the strongest demographic characteristics associated with infant sleep were maternal schooling, family income, TV watching, and having other children in the home. Furthermore, the evidence suggests that certain sleep parameters may be more influenced by environmental characteristics than others, such as, for example, bed and wake time, and co-sleeping.

Younger maternal age was associated with shorter sleep duration, with later waketimes and higher rates of co-sleeping. This is in line with other studies we identified in the literature where maternal age (>35 years) was consistently associated with shorter sleep duration [15]. Maternal schooling years (>9) and higher family income were consistently and positively associated with later bed and wake times, whereas lower maternal education and income were associated with higher levels of co-sleeping. A gender effect was also observed with girls waking up later, a finding consistent with data from the UK [27]. Boys were more likely to wake up at night and to sleep longer in the day. Increased TV-watching at nighttime was associated with shorter total and nighttime sleep duration, a finding also previously reported in the literature [27]. Increased TV-watching at nighttime was also associated with later bed and wake times and an increased rate of sleep disturbances, a finding that has previously been reported in children of similar age [28], [29]. It is notable/noteworthy that although TV watching at nighttime may contribute to more sleep disturbances, it is also possible that parents of children already experiencing difficulties with falling asleep use TV as a way to distract or calm down children before bedtime.

Co-sleeping was associated with earlier bed and wake times, more awakenings and sleeping longer in the day. Breastfeeding was associated with increased awakenings and higher rates of co-sleeping. In this sample, maternal skin color was consistently associated with co-sleeping, with the lowest rates of co-sleeping among white women. Finally, having other children in the home was associated with earlier bed and wake times, shorter sleep duration at night, and an increased risk of sleep disturbances in comparison with participants without other children in the home.

Previous awakenings were an important predictor of later awakenings both at 24 and 48 months, which is in line with previous research reporting previous awakenings as significant predictors of later awakenings [14].

It is difficult to draw conclusions on what is optimal and recommended sleep. Recommendations will need to take into account a number of issues other than mean values including overall quality of sleep and whether the child displays signs of sleepiness or does not wake up spontaneously in the morning [30]. Longer sleep duration or no awakenings cannot invariably be interpreted as optimal sleep. Moreover, it is important to consider that sleep is an appetitive behavior where wanting more is not indicative of needing more sleep but may indicate a choice for more sleep [31], similar to behaviors like eating or drinking.

This study has some important strengths. We utilized a large prospective cohort to describe sleep characteristics in a normative population in Pelotas, Brazil. The cohort had very little attrition throughout the time points (<7%) and this is important as parents of children with significant sleep disturbances may find parenting more challenging and may be less likely to participate in research which could lead to biased estimates of sleep characteristics. Nevertheless, in terms of sociodemographic characteristics, we report some differences in terms of attrition. The included sample had a higher proportion of families with higher income, higher maternal education, and lower levels of maternal smoking. One major limitation is that sleep data was collected through maternal report. This is a common limitation of large cohorts, particularly as self-reporting is not available with young children and the cost of more objective measures is often prohibitive. As most other cohorts discussed in this paper also relied on maternal reports of infant sleep, comparison across cohorts is more analogous. Studies specifically examining agreement between parental questionnaires and actigraphy, a more objective measure of rest and activity patterns, report acceptable agreement, for example, for bedtime (±28 min), waketime (±24 min) and for sleep duration (±32 min) [32], [33]. Overall parental reports tend to over-rather than underestimate bedtime and sleep duration and to underestimate night awakenings compared to actigraphy. Parental reports of variables such as awakenings may be more biased, because parents will only be aware of these behaviors if the child seeks parental attention, and the literature reports moderate to large discrepancies [34] with under 10% of awakenings recorded on actigraphy being reported by parental report [32]. Discrepancies between maternal and objective reports will need to be taken into account, particularly when comparing sleep data collected using different methodologies, however, the large longitudinal studies offering reference means for children's sleep development have overwhelmingly used maternal reports and therefore our findings remain comparable to these [13], [15], [22], [35]. Parental reports of sleep also present with some advantages as parents have greater insight in behaviors that may not manifest daily, and may not be captured in short-term (objective) recordings. Parental perceptions of sleep, particularly when sleep is perceived as problematic, are important when children present to pediatricians with potential sleep difficulties as doctors rely on parental reports. The Pelotas cohort is drawn from an urban medium-sized city in the south of Brazil, and thus findings may not generalize to other populations such as rural samples. The data are part of a birth cohort with very rich prospectively collected longitudinal data, starting 12 years ago. Analysis of historic trends in sleep in older children (5–18 years of age) indicates small changes in variables such as duration with a decrease of about 0.75 min per year [17], suggesting potential changes of very small magnitude in the estimates of sleep parameters from this cohort (between 6 and 9 min).

Future work should investigate how later bed/wake times contribute to child development in domains such as day-to-day functioning, cognitive development, and emotional and behavioral development. This large cohort provides evidence of sleep patterns which are substantially different in a Brazilian community sample compared to published data from Europe and the USA, with a 2-h shift in the bedtime and high rates of co-sleeping, across the first few years of life, whilst other sleep parameters remain mostly similar. These data challenge our understanding of optimal sleep patterns including the clinical guidelines in HICs which argue for initiating bedtime patterns around 20:00. Our study provides information on the inter-individual and intra-individual variations in sleep characteristics and contributes to what constitutes ‘normal’ sleep by providing data from a large prospective and representative urban community sample in Brazil.

## Author contributions

EN conducted the data analysis, was involved in the interpretation of results and the preparation of the manuscript. IS was involved in data collection, the interpretation of results and the preparation of the manuscript. AS was involved in the interpretation of results and the preparation of the manuscript. FB was involved in the study design. AB was involved in the study design, data collection and preparation of the manuscript. AM was involved in the study design and data collection. She was also involved in the data analysis, interpretation of the results and preparation of the manuscript. All authors approved the final version of the manuscript. EN and AM will serve as guarantors for the contents of this paper.
