# Supplementary material for: A different rhythm of life: sleep patterns in the first 4 years of life and associated sociodemographic characteristics in a large Brazilian birth cohort
Source: Sleep Med. 2017 Sep;37:77–87. doi: 10.1016/j.sleep.2017.06.001 (PMC5609565; doi:10.1016/j.sleep.2017.06.001)
Supplement: Supplementary file 1 [file mmc1.docx]

Supplementary tables

Table S1. Description of the sample.

|  |  | Participants with sleep data available  *N*=3842  *N* (%) or  mean (SD) | Participants with no sleep data available *N*=309  *N* (%) or  mean (SD) | Test statistic |
| --- | --- | --- | --- | --- |
| Family income quintiles | 1 | 766 (19.9) | 87 (28.2) | *p*<0.001 |
|  | 2 | 764 (19.9) | 76 (24.6) |  |
|  | 3 | 767 (20.0) | 43 (13.9) |  |
|  | 4 | 809 (21.1) | 43 (13.9) |  |
|  | 5 | 736 (19.2) | 60 (19.4) |  |
| Maternal schooling (years) | 0–4 | 582 (15.1) | 60 (19.4) | *p*=0.015 |
|  | 5–8 | 1575 (41.0) | 138 (44.7) |  |
|  | ≥9 | 1685 (43.9) | 111 (35.9) |  |
| Maternal age (years) | ≤19 | 719 (18.7) | 71 (23.0) | *p*=0.093 |
|  | 20-34 | 2610 (679) | 206 (66.7) |  |
|  | ≥35 | 513 (13.4) | 32 (10.4) |  |
| Maternal skin color | White | 2810 (73.1) | 218 (70.6) | *p*=0.606 |
|  | Black | 763 (19.9) | 68 (22.0) |  |
|  | Other | 269 (7.0) | 23 (7.4) |  |
| Parity | 1 | 1519 (39.5) | 118 (38.2) | *p*=0.684 |
|  | 2 | 1007 (26.2) | 88 (28.5) |  |
|  | ≥3 | 1316 (34.3) | 103 (33.3) |  |
| Smoked during pregnancy | No | 2870 (74.7) | 214 (69.3) | *p*=0.035 |
| Consumed alcohol during pregnancy | No | 3724 (96.9) | 292 (94.5) | *p*=0.021 |
| Child gender | Male | 1998 (48) | 164 (53.1) | *p*=0.717 |
| Gestational age at birth (weeks) | ≤28 | 9 (0.2) | 22 (7.1) | *p*<0.001 |
|  | 29–32 | 60 (1.6) | 12 (3.9) |  |
|  | 33–36 | 843 (21.9) | 78 (25.2) |  |
|  | ≥37 | 2930 (76.3) | 197 (63.8) |  |
| Birth weight | ≥2500 g | 3508 (91.3) | 241 (78) | *p*<0.001 |
| Neonatal complications | APGAR score≥7 | 3779 (98.4) | 284 (91.9) | *p*<0.001 |
| Mode of delivery | Cesarean | 1741 (45.3) | 138 (44.7) | *p*=0.824 |
| Breastfeeding – 3 months | Yes | 2696 (92) |  |  |
| Breastfeeding – 12 months | No | 2266 (59) |  |  |
|  | Yes | 1426 (37.1) |  |  |
|  | Never breastfed | 150 (3.9) |  |  |
| Breastfeeding – 24 months | No | 2796 (73.6) |  |  |
|  | Yes | 838 (22.1) |  |  |
|  | Never breastfed | 165 (4.3) |  |  |
| Co-sleeping (yes) | 3 months | 1888 (49.5) |  |  |
|  | 12 months | 1765 (48.8) |  |  |
|  | 24 months | 1840 (51.5) |  |  |
|  | 48 months | 1701 (52.2) |  |  |
| TV watching total (min, mean (SD)) | 24 months | 110.59 (100.82) |  |  |
|  | 48months | 203.32 (130.38) |  |  |
| TV watching nighttime (min, mean (SD)) | 24 months | 42.11 (51.61) |  |  |
|  | 48 months | 74.36 (64.22) |  |  |
| Younger children at home (yes) | 24 months | 322 (8.54) |  |  |
|  | 48months | 795 (21.27) |  |  |
| Older children at home (yes) | 24 months | 1493 (39.6) |  |  |
|  | 48 months | 1219 (32.62) |  |  |

SD, standard deviation.

Table S2. Hierarchical linear regression analysis for total sleep duration and associated sociodemographics characteristics from 3 to 48 months.

|  | **3 months**  ***N*=823** | | | **12 months**  ***N*=3578** | | | **24 months**  ***N*=3046** | | | **48 months**  ***N*=2809** | | |
| --- | --- | --- | --- | --- | --- | --- | --- | --- | --- | --- | --- | --- |
|  | **B** | **95% CI** | | **B** | **95% CI** | | **B** | **95% CI** | | **B** | **95% CI** | |
|  |  | **Lower** | **Upper** |  | **Lower** | **Upper** |  | **Lower** | **Upper** |  | **Lower** | **Upper** |
| Maternal characteristics |  |  |  |  |  |  |  |  |  |  |  |  |
| Maternal skin color |  |  |  |  |  |  |  |  |  |  |  |  |
| White | -0.1 | -0.47 | 0.26 | 0.04 | -0.07 | 0.14 | -0.02 | -0.11 | 0.06 | -0.01 | -0.05 | 0.03 |
| Black | -0.1 | -0.46 | 0.26 | 0 | -0.11 | 0.1 | -0.05 | -0.13 | 0.04 | 0 | -0.05 | 0.04 |
| Other | REF |  |  | REF |  |  | REF |  |  | REF |  |  |
| Maternal age (years) |  |  |  |  |  |  |  |  |  |  |  |  |
| ≤19 | 0.19 | -0.15 | 0.53 | 0.06 | -0.04 | 0.16 | 0.03 | -0.05 | 0.11 | -0.01 | -0.05 | 0.03 |
| 20–34 | 0.18 | -0.13 | 0.49 | 0.03 | -0.06 | 0.12 | -0.01 | -0.08 | 0.06 | 0.02 | -0.01 | 0.06 |
| ≥35 | REF |  |  | REF |  |  | REF |  |  | REF |  |  |
| Smoked during pregnancy (no) | -0.07 | -0.55 | 0.41 | 0.05 | -0.01 | 0.11 | 0.02 | -0.03 | 0.06 | 0.01 | -0.02 | 0.03 |
| Alcohol during pregnancy (no) | -0.02 | -0.22 | 0.17 | 0 | -0.06 | 0.06 | -0.01 | -0.06 | 0.04 | 0 | -0.03 | 0.02 |
| Maternal schooling (years) |  |  |  |  |  |  |  |  |  |  |  |  |
| 0–4 | -0.11 | -0.38 | 0.16 | -0.07 | -0.14 | 0.01 | -0.02 | -0.08 | 0.04 | 0.02 | -0.01 | 0.05 |
| 5–8 | -0.12 | -0.37 | 0.13 | -0.08 | -0.16 | -0.01 | 0.05 | -0.01 | 0.11 | 0.03 | 0.00 | 0.06 |
| ≥9 | REF |  |  | REF |  |  | REF |  |  | REF |  |  |
| Parity |  |  |  |  |  |  |  |  |  |  |  |  |
| 1 | -0.11 | -0.41 | 0.19 | 0.05 | -0.04 | 0.13 | -0.05 | -0.14 | 0.03 | 0.01 | -0.03 | 0.05 |
| 2 | 0.02 | -0.24 | 0.27 | 0.02 | -0.05 | 0.1 | -0.02 | -0.08 | 0.04 | 0.01 | -0.01 | 0.04 |
| ≥3 | REF |  |  | REF |  |  | REF |  |  | REF |  |  |
| Family income quintiles |  |  |  |  |  |  |  |  |  |  |  |  |
| 1 | -0.08 | -0.36 | 0.21 | 0.03 | -0.06 | 0.12 | 0.05 | -0.02 | 0.12 | 0.01 | -0.02 | 0.05 |
| 2 | -0.11 | -0.38 | 0.16 | -0.05 | -0.14 | 0.03 | 0.05 | -0.02 | 0.12 | 0.00 | -0.03 | 0.04 |
| 3 | 0.17 | -0.11 | 0.44 | 0.01 | -0.07 | 0.09 | 0.07 | 0.01 | 0.14 | 0.00 | -0.04 | 0.03 |
| 4 | 0.01 | -0.24 | 0.26 | 0.01 | -0.07 | 0.09 | 0.06 | -0.01 | 0.12 | 0.00 | -0.03 | 0.03 |
| 5 | REF |  |  | REF |  |  | REF |  |  | REF |  |  |
| Child characteristics |  |  |  |  |  |  |  |  |  |  |  |  |
| Child gender (Male) | -0.12 | -0.32 | 0.08 | -0.02 | -0.08 | 0.04 | -0.03 | -0.08 | 0.02 | 0.02 | 0.00 | 0.05 |
| Gestational age at birth (weeks) |  |  |  |  |  |  |  |  |  |  |  |  |
| ≤28 | 0.24 | -0.12 | 0.6 | 0.05 | -0.06 | 0.16 | 0.02 | -0.07 | 0.12 | -0.12 | -0.63 | 0.4 |
| 29–32 | 0.07 | -0.13 | 0.27 | -0.07 | -0.14 | 0 | 0.03 | -0.03 | 0.09 | -0.09 | -0.3 | 0.11 |
| 33–36 | -0.02 | -0.23 | 0.2 | -0.07 | -0.13 | -0.01 | -0.01 | -0.06 | 0.05 | 0.02 | -0.04 | 0.08 |
| ≥37 | REF |  |  | REF |  |  | REF |  |  | REF |  |  |
| Birth weight (g) <2500 | 0.19 | -0.07 | 0.44 | 0.00 | -0.07 | 0.07 | -0.03 | -0.08 | 0.03 | -0.01 | -0.04 | 0.02 |
| Neonatal complications (APGAR<7) | 0.04 | -0.17 | 0.24 | 0.01 | -0.05 | 0.08 | 0.01 | -0.04 | 0.07 | 0.01 | -0.01 | 0.04 |
| Environmental/other factors |  |  |  |  |  |  |  |  |  |  |  |  |
| Mode of Delivery (normal) | 0.02 | -0.19 | 0.23 | 0.01 | -0.05 | 0.07 | -0.04 | -0.1 | 0.01 | 0.02 | -0.01 | 0.04 |
| Co-sleeping (yes) | -0.19 | -0.41 | 0.04 | 0.00 | -0.07 | 0.07 | -0.04 | -0.09 | 0.01 | 0.00 | -0.02 | 0.03 |
| Breastfeeding (no) | 0.06 | -0.14 | 0.26 | 0.13 | -0.03 | 0.28 | 0.08 | -0.03 | 0.19 |  |  |  |
| Yes | REF |  |  | 0.07 | -0.08 | 0.23 | 0.08 | -0.03 | 0.19 |  |  |  |
| Never breastfed |  |  |  | REF |  |  | REF |  |  |  |  |  |
| TV watching total |  |  |  |  |  |  | 0.03 | -0.03 | 0.09 | -0.01 | -0.03 | 0.02 |
| TV at nighttime |  |  |  |  |  |  | 0.01 | -0.04 | 0.07 | -0.06** | -0.08 | -0.03 |
| Younger children at home (yes) |  |  |  |  |  |  | 0.09** | 0.04 | 0.14 | 0.02 | -0.01 | 0.04 |
| Older children at home (yes) |  |  |  |  |  |  | -0.12** | -0.18 | -0.05 | 0.00 | -0.03 | 0.03 |
|  | R^2^=0.02, F(23,799)=0.81 | | | R^2^=0.01, F(23,3563)=1.94 | | | R^2^=0.021, F(28,3017)=2.26 | | | R^2^=0.02, F(26,2782)=2.11 | | |

CI, confidence interval.

**p*<0.01, ***p*≤0.001.

Table S3. Hierarchical linear regression analysis for sleep duration during the day and associated sociodemographic characteristics from 3 to 48 months.

|  | **3 months**  ***N*=817** | | | **12 months**  ***N*=3583** | | | **24 months**  ***N*=3049** | | | **48 months**  ***N*=3398** | | |
| --- | --- | --- | --- | --- | --- | --- | --- | --- | --- | --- | --- | --- |
|  | **B** | **95% CI** | | **B** | **95% CI** | | **B** | **95% CI** | | **B** | **95% CI** | |
|  |  | **Lower** | **Upper** |  | **Lower** | **Upper** |  | **Lower** | **Upper** |  | **Lower** | **Upper** |
| Maternal characteristics |  |  |  |  |  |  |  |  |  |  |  |  |
| Maternal skin color |  |  |  |  |  |  |  |  |  |  |  |  |
| White | -0.17 | -0.47 | 0.13 | 0.03 | -0.05 | 0.1 | -0.03 | -0.09 | 0.03 | -0.05 | -0.1 | 0 |
| Black | -0.17 | -0.46 | 0.13 | 0 | -0.08 | 0.08 | -0.04 | -0.1 | 0.02 | 0.02 | -0.03 | 0.07 |
| Other | REF |  |  | REF |  |  | REF |  |  | REF |  |  |
| Maternal age (years) |  |  |  |  |  |  |  |  |  |  |  |  |
| ≤19 | 0.1 | -0.18 | 0.38 | -0.04 | -0.12 | 0.03 | -0.05 | -0.11 | 0 | 0 | -0.05 | 0.05 |
| 20–34 | 0.11 | -0.15 | 0.36 | -0.03 | -0.1 | 0.03 | -0.03 | -0.08 | 0.02 | 0.02 | -0.02 | 0.06 |
| ≥35 | REF |  |  | REF |  |  | REF |  |  | REF |  |  |
| Smoked during pregnancy (no) | 0 | -0.4 | 0.39 | 0.04 | -0.01 | 0.08 | 0.01 | -0.02 | 0.04 | 0.01 | -0.02 | 0.04 |
| Alcohol during pregnancy (no) | 0.03 | -0.13 | 0.19 | -0.02 | -0.07 | 0.02 | 0.01 | -0.02 | 0.04 | 0 | -0.03 | 0.03 |
| Maternal schooling (years) |  |  |  |  |  |  |  |  |  |  |  |  |
| 0–4 | -0.22 | -0.44 | 0.01 | -0.04 | -0.09 | 0.02 | 0.02 | -0.02 | 0.06 | 0.01 | -0.03 | 0.05 |
| 5–8 | -0.07 | -0.28 | 0.14 | -0.05 | -0.1 | 0.01 | 0.02 | -0.02 | 0.06 | 0.02 | -0.02 | 0.05 |
| ≥9 | REF |  |  | REF |  |  | REF |  |  | REF |  |  |
| Parity |  |  |  |  |  |  |  |  |  |  |  |  |
| 1 | -0.22 | -0.47 | 0.03 | 0.03 | -0.03 | 0.09 | 0.01 | -0.05 | 0.06 | -0.01 | -0.06 | 0.04 |
| 2 | 0 | -0.21 | 0.21 | 0 | -0.05 | 0.06 | 0.02 | -0.02 | 0.06 | 0.01 | -0.02 | 0.05 |
| ≥3 | REF |  |  | REF |  |  | REF |  |  | REF |  |  |
| Family income quintiles |  |  |  |  |  |  |  |  |  |  |  |  |
| 1 | -0.22 | -0.45 | 0.02 | -0.01 | -0.07 | 0.06 | 0.03 | -0.02 | 0.08 | 0.03 | -0.02 | 0.07 |
| 2 | -0.19 | -0.42 | 0.03 | -0.06 | -0.12 | 0.01 | 0.03 | -0.01 | 0.08 | 0.06* | 0.01 | 0.1 |
| 3 | 0.09 | -0.14 | 0.32 | -0.03 | -0.1 | 0.03 | 0.05 | 0 | 0.09 | 0.03 | -0.01 | 0.07 |
| 4 | -0.08 | -0.28 | 0.13 | -0.03 | -0.09 | 0.03 | 0.04 | 0 | 0.09 | 0.03 | 0 | 0.07 |
| 5 | REF |  |  | REF |  |  | REF |  |  | REF |  |  |
| Child characteristics |  |  |  |  |  |  |  |  |  |  |  |  |
| Child gender (male) | -0.13 | -0.3 | 0.04 | -0.05 | -0.1 | -0.01 | -0.06** | -0.09 | -0.02 | -0.02 | -0.05 | 0.01 |
| Gestational age at birth (weeks) |  |  |  |  |  |  |  |  |  |  |  |  |
| ≤28 | -0.15 | -0.57 | 0.26 | 0.04 | -0.04 | 0.12 | -0.02 | -0.08 | 0.04 | 0.03 | -0.68 | 0.74 |
| 29–32 | 0.11 | -0.06 | 0.27 | -0.04 | -0.09 | 0.01 | -0.02 | -0.06 | 0.02 | -0.06 | -0.32 | 0.21 |
| 33–36 | 0.14 | -0.04 | 0.31 | -0.03 | -0.08 | 0.01 | -0.02 | -0.05 | 0.02 | 0 | -0.07 | 0.07 |
| ≥37 | REF |  |  | REF |  |  | REF |  |  | REF |  |  |
| Birth weight (g) <2500 | 0.19 | -0.02 | 0.4 | 0.02 | -0.03 | 0.07 | -0.01 | -0.05 | 0.03 | -0.02 | -0.06 | 0.01 |
| Neonatal complications (APGAR<7) | 0.03 | -0.14 | 0.2 | -0.01 | -0.07 | 0.04 | -0.02 | -0.06 | 0.02 | 0.02 | -0.01 | 0.05 |
| Environmental/other factors |  |  |  |  |  |  |  |  |  |  |  |  |
| Mode of delivery (normal) | 0.07 | -0.1 | 0.25 | 0 | -0.04 | 0.05 | 0 | -0.03 | 0.04 | -0.01 | -0.04 | 0.02 |
| Co-sleeping (yes) | -0.01 | -0.2 | 0.17 | -0.05 | -0.1 | 0 | 0.01 | -0.02 | 0.04 | -0.06* | -0.09 | -0.03 |
| Breastfeeding (no) | -0.05 | -0.22 | 0.12 | 0.08 | -0.04 | 0.19 | 0.09 | 0.01 | 0.16 |  |  |  |
| Breastfeeding (yes) | REF |  |  | 0.02 | -0.1 | 0.13 | 0.02 | -0.05 | 0.09 |  |  |  |
| Never breastfed |  |  |  | REF |  |  | REF |  |  |  |  |  |
| TV watching total |  |  |  |  |  |  | 0.03 | -0.01 | 0.06 | -0.02 | -0.05 | 0.02 |
| TV at nighttime |  |  |  |  |  |  | 0.00 | -0.05 | 0.05 | 0.03 | 0.00 | 0.06 |
| Younger children at home (yes) |  |  |  |  |  |  | 0.00 | -0.03 | 0.03 | -0.02 | -0.05 | 0.01 |
| Older children at home (yes) |  |  |  |  |  |  | -0.05 | -0.1 | -0.01 | -0.03 | -0.06 | 0.01 |
|  | R^2^=0.04, F(23,814)=1.53 | | | R^2^=0.02, F(24,3558)=2.33 | | | R^2^=0.02, F(28,3020)=1.97 | | | R^2^=0.02, F(26,3371)=2.73 | | |
|  |  | | |  | | |  | | |  | | |

CI, confidence interval.

**p*<0.01, ***p*≤0.001.

Table S4. Hierarchical linear regression analysis for nighttime sleep duration and associated sociodemographic characteristics from 3 to 48 months.

|  | **3 months**  ***N*=838** | | | **12 months**  ***N*=3588** | | | **24 months**  ***N*=3056** | | | **48 months**  ***N*=3423** | | |
| --- | --- | --- | --- | --- | --- | --- | --- | --- | --- | --- | --- | --- |
|  | **B** | **95% CI** | | **B** | **95% CI** | | **B** | **95% CI** | | **B** | **95% CI** | |
|  |  | **Lower** | **Upper** |  | **Lower** | **Upper** |  | **Lower** | **Upper** |  | **Lower** | **Upper** |
| Maternal characteristics |  |  |  |  |  |  |  |  |  |  |  |  |
| Maternal skin color |  |  |  |  |  |  |  |  |  |  |  |  |
| White | 0.15 | -0.06 | 0.35 | 0.01 | -0.06 | 0.09 | -0.01 | -0.08 | 0.07 | 0.04 | -0.03 | 0.11 |
| Black | 0.14 | -0.06 | 0.35 | -0.02 | -0.1 | 0.06 | -0.03 | -0.11 | 0.04 | -0.02 | -0.09 | 0.05 |
| Other | REF |  |  | REF |  |  | REF |  |  | REF |  |  |
| Maternal age (years) |  |  |  |  |  |  |  |  |  |  |  |  |
| ≤19 | 0.01 | -0.18 | 0.21 | 0.11* | 0.04 | 0.18 | 0.1* | 0.03 | 0.17 | -0.02 | -0.09 | 0.05 |
| 20–34 | 0.05 | -0.12 | 0.23 | 0.07 | 0 | 0.13 | 0.03 | -0.04 | 0.09 | 0.02 | -0.04 | 0.08 |
| ≥35 | REF |  |  | REF |  |  | REF |  |  | REF |  |  |
| Smoked during pregnancy (no) | -0.06 | -0.33 | 0.21 | 0.01 | -0.03 | 0.06 | 0 | -0.05 | 0.04 | 0.01 | -0.03 | 0.05 |
| Alcohol during pregnancy (no) | -0.06 | -0.17 | 0.04 | 0.03 | -0.02 | 0.07 | -0.03 | -0.08 | 0.01 | 0.01 | -0.03 | 0.05 |
| Maternal schooling (years) |  |  |  |  |  |  |  |  |  |  |  |  |
| 0–4 | 0.01 | -0.14 | 0.17 | -0.02 | -0.08 | 0.03 | -0.03 | -0.08 | 0.03 | 0 | -0.05 | 0.05 |
| 5–8 | -0.09 | -0.23 | 0.06 | -0.03 | -0.08 | 0.02 | 0.02 | -0.04 | 0.07 | 0.03 | -0.02 | 0.08 |
| ≥9 | REF |  |  | REF |  |  | REF |  |  | REF |  |  |
| Parity |  |  |  |  |  |  |  |  |  |  |  |  |
| 1 | -0.03 | -0.2 | 0.15 | 0.04 | -0.02 | 0.1 | -0.05 | -0.13 | 0.02 | -0.01 | -0.07 | 0.06 |
| 2 | -0.09 | -0.23 | 0.06 | 0.04 | -0.02 | 0.09 | -0.03 | -0.08 | 0.02 | 0.01 | -0.04 | 0.06 |
| ≥3 | REF |  |  | REF |  |  | REF |  |  | REF |  |  |
| Family income quintiles |  |  |  |  |  |  |  |  |  |  |  |  |
| 1 | 0.14 | -0.03 | 0.3 | 0.03 | -0.03 | 0.1 | 0.03 | -0.03 | 0.09 | 0.04 | -0.02 | 0.1 |
| 2 | -0.02 | -0.17 | 0.14 | 0.01 | -0.05 | 0.07 | 0.02 | -0.04 | 0.08 | -0.02 | -0.08 | 0.04 |
| 3 | 0.04 | -0.11 | 0.2 | 0.05 | -0.01 | 0.1 | 0.03 | -0.03 | 0.08 | 0 | -0.05 | 0.05 |
| 4 | 0.06 | -0.08 | 0.21 | 0.05 | -0.01 | 0.11 | 0.02 | -0.04 | 0.07 | 0 | -0.05 | 0.05 |
| 5 | REF |  |  | REF |  |  | REF |  |  | REF |  |  |
| Child characteristics |  |  |  |  |  |  |  |  |  |  |  |  |
| Child gender (male) | 0.05 | -0.07 | 0.16 | 0.02 | -0.02 | 0.06 | 0.03 | -0.01 | 0.07 | 0.07** | 0.03 | 0.11 |
| Gestational age at birth (weeks) |  |  |  |  |  |  |  |  |  |  |  |  |
| ≤28 | -0.19 | -0.4 | 0.02 | 0.02 | -0.06 | 0.1 | 0.11* | 0.03 | 0.18 | 0.27 | -0.63 | 1.17 |
| 29–32 | -0.02 | -0.14 | 0.09 | -0.03 | -0.08 | 0.02 | 0.03 | -0.02 | 0.08 | -0.05 | -0.4 | 0.3 |
| 33–36 | -0.13 | -0.25 | 0 | -0.02 | -0.07 | 0.02 | 0.01 | -0.04 | 0.05 | 0 | -0.11 | 0.1 |
| ≥37 | REF |  |  | REF |  |  | REF |  |  | REF |  |  |
| Birth weight (g) <2500 | 0.06 | -0.09 | 0.21 | -0.04 | -0.1 | 0.01 | -0.02 | -0.07 | 0.03 | -0.04 | -0.08 | 0.01 |
| Neonatal complications (APGAR <7) | 0.01 | -0.11 | 0.13 | 0.01 | -0.04 | 0.06 | 0.02 | -0.02 | 0.07 | 0.01 | -0.04 | 0.05 |
| Environmental/other factors |  |  |  |  |  |  |  |  |  |  |  |  |
| Mode of delivery (normal) | 0.02 | -0.1 | 0.14 | -0.02 | -0.06 | 0.03 | -0.06* | -0.11 | -0.02 | 0.04 | 0 | 0.08 |
| Co-sleeping (yes) | -0.18* | -0.3 | -0.05 | 0.07* | 0.02 | 0.11 | 0.03 | -0.2 | 0.07 | 0.00 | -0.04 | 0.04 |
| Breastfeeding (no) | 0.1 | -0.02 | 0.22 | -0.01 | -0.12 | 0.11 | 0.01 | -0.08 | 0.1 |  |  |  |
| Breastfeeding (yes) | REF |  |  | 0 | -0.11 | 0.12 | 0.08 | -0.02 | 0.17 |  |  |  |
| Never breastfed |  |  |  | REF |  |  | REF |  |  |  |  |  |
| TV watching total |  |  |  |  |  |  | 0.01 | -0.04 | 0.06 | 0.01 | -0.03 | 0.05 |
| TV at nighttime |  |  |  |  |  |  | 0.00 | -0.04 | 0.04 | -0.13** | -0.18 | -0.09 |
| Younger children at home (yes) |  |  |  |  |  |  | 0.08** | 0.03 | 0.12 | 0.06* | 0.02 | 0.1 |
| Older children at home (yes) |  |  |  |  |  |  | -0.04 | -0.09 | 0.02 | 0.03 | -0.02 | 0.08 |
|  | R^2^=0.04, F(23,814)=1.39 | | | R^2^=0.01, F(24,3563)=1.95 | | | R^2^=0.02, F(28,3027)=2.43 | | | R^2^=0.03, F(26,3396)=3.73 | | |
| CI, confidence interval.  **p*<0.01, ***p*≤0.001. |  | | |  | | |  | | |  | | |

Table S5. Poisson regression analysis of co-sleeping and sociodemographic characteristics from 3 to 48 months.

|  | 3 months  *N*=3660 | | | 12 months  *N*=3617 | | | | | | | 24 months  *N*=2903 | | | 48 months  N=2,988 | | |
| --- | --- | --- | --- | --- | --- | --- | --- | --- | --- | --- | --- | --- | --- | --- | --- | --- |
|  | B | 95% CI | | B | | 95% CI | | | | | B | 95% CI | | B | 95% CI | |
|  |  | **Lower** | **Upper** |  | | **Lower** | | | | **Upper** |  | **Lower** | **Upper** |  | **Lower** | **Upper** |
| Maternal Ccharacteristics |  |  |  |  | |  | | | |  |  |  |  |  |  |  |
| Maternal skin color |  |  |  |  | |  | | | |  |  |  |  |  |  |  |
| White | 0.89** | 0.75 | 1.06 | 0.80** | | 0.68 | | | | 0.95 | 0.87** | 0.72 | 1.05 | 0.94* | 0.77 | 1.14 |
| Black | 1.13 | 0.94 | 1.36 | 1.06 | | 0.88 | | | | 1.27 | 1.09 | 0.89 | 1.33 | 1.13 | 0.92 | 1.39 |
| Other | 1.00 |  |  | 1.00 | |  | | | |  | 1.00 |  |  | 1.00 |  |  |
| Maternal age (years) |  |  |  |  | |  | | | |  |  |  |  |  |  |  |
| ≤19 | 1.19 | 0.98 | 1.45 | 1.52** | | 1.25 | | | | 1.86 | 1.50** | 1.20 | 1.87 | 1.11 | 0.90 | 1.36 |
| 20–34 | 1.08 | 0.93 | 1.25 | 1.23 | | 1.06 | | | | 1.44 | 1.25 | 1.05 | 1.48 | 1.03 | 0.88 | 1.20 |
| ≥35 | 1.00 |  |  | 1.00 | |  | | | |  | 1.00 |  |  | 1.00 |  |  |
| Smoked during pregnancy |  |  |  |  | |  | | | |  |  |  |  |  |  |  |
| No | 0.92 | 0.83 | 1.02 | 1.01 | | 0.90 | | | | 1.12 | 0.92 | 0.82 | 1.03 | 0.92 | 0.82 | 1.03 |
| Yes | 1.00 |  |  | 1.00 | |  | | | |  | 1.00 |  |  | 1.00 |  |  |
| Alcohol during pregnancy |  |  |  |  | |  | | | |  |  |  |  |  |  |  |
| No | 0.93 | 0.73 | 1.18 | 0.95 | | 0.73 | | | | 1.23 | 0.86 | 0.66 | 1.13 | 0.83 | 0.64 | 1.08 |
| Yes | 1.00 |  |  | 1.00 | |  | | | |  | 1.00 |  |  | 1.00 |  |  |
| Maternal schooling (years) |  |  |  |  | |  | | | |  |  |  |  |  |  |  |
| 0-4 | 0.92** | 0.81 | 1.06 | 1.34** | | 1.15 | | | | 1.56 | 1.21 | 1.02 | 1.43 | 1.07 | 0.91 | 1.26 |
| 5–8 | 0.91 | 0.80 | 1.03 | 1.21 | | 1.07 | | | | 1.36 | 1.08 | 0.95 | 1.24 | 1.00 | 0.88 | 1.13 |
| ≥9 | 1.00 |  |  | 1.00 | |  | | | |  | 1.00 |  |  | 1.00 |  |  |
| Parity |  |  |  |  | |  | | | |  |  |  |  |  |  |  |
| 1 | 1.08 | 0.95 | 1.24 | 0.85 | | 0.74 | | | | 0.97 | 0.85 | 0.73 | 0.98 | 0.83 | 0.71 | 0.95 |
| 2 | 0.98 | 0.86 | 1.12 | 0.88 | | 0.78 | | | | 1.00 | 0.82 | 0.71 | 0.94 | 0.84 | 0.74 | 0.96 |
| ≥3 | 1.00 |  |  | 1.00 | |  | | | |  | 1.00 |  |  | 1.00 |  |  |
| Family income quintiles |  |  |  |  | |  | | | |  |  |  |  |  |  |  |
| 1 | 1.51** | 1.27 | 1.81 | 1.54** | | 1.28 | | | | 1.85 | 1.39** | 1.15 | 1.69 | 1.52* | 1.25 | 1.84 |
| 2 | 1.41 | 1.18 | 1.69 | 1.49 | | 1.24 | | | | 1.80 | 1.27 | 1.05 | 1.54 | 1.32 | 1.09 | 1.60 |
| 3 | 1.28 | 1.07 | 1.53 | 1.36 | | 1.14 | | | | 1.64 | 1.19 | 0.98 | 1.44 | 1.26 | 1.04 | 1.53 |
| 4 | 1.16 | 0.97 | 1.39 | 1.12 | | 0.93 | | | | 1.35 | 0.98 | 0.81 | 1.19 | 1.20 | 0.99 | 1.45 |
| 5 | 1.00 |  |  | 1.00 | |  | | | |  | 1.00 |  |  | 1.00 |  |  |
| Child characteristics |  |  |  |  | |  | | | |  |  |  |  |  |  |  |
| Child gender |  |  |  |  | |  | | | |  |  |  |  |  |  |  |
| Male | 1.01 | 0.92 | 1.10 | 1.06 | | 0.96 | | | | 1.16 | 1.09 | 0.98 | 1.21 | 1.01 | 0.91 | 1.12 |
| Female | 1.00 |  |  | 1.00 | |  | | | |  | 1.00 |  |  | 1.00 |  |  |
| Gestational age at birth (weeks) |  |  |  |  | |  | | | |  |  |  |  |  |  |  |
| ≤28 | 0.22 | 0.03 | 1.57 | 0.54 | | 0.13 | | | | 2.20 | 0.35 | 0.05 | 2.50 | 1.08 | 0.40 | 2.95 |
| 29–32 | 0.85 | 0.58 | 1.25 | 1.16 | | 0.80 | | | | 1.69 | 1.30 | 0.88 | 1.92 | 0.93 | 0.61 | 1.41 |
| 33–36 | 1.09 | 0.97 | 1.22 | 1.04 | | 0.92 | | | | 1.16 | 1.04 | 0.92 | 1.18 | 0.96 | 0.85 | 1.09 |
| ≥37 | 1.00 |  |  | 1.00 | |  | | | |  | 1.00 |  |  | 1.00 |  |  |
| Birth weight (g) |  |  |  |  | |  | | | |  |  |  |  |  |  |  |
| <2500 | 1.15 | 0.98 | 1.37 | 1.07 | | 0.89 | | | | 1.28 | 1.02 | 0.83 | 1.24 | 1.05 | 0.87 | 1.27 |
| ≥2500 | 1.00 |  |  | 1.00 | |  | | | |  | 1.00 |  |  | 1.00 |  |  |
| Neonatal complications (APGAR score) |  |  |  |  | |  | | | |  |  |  |  |  |  |  |
| <7 | 0.98 | 0.68 | 1.42 | 1.05 | | 0.73 | | | | 1.51 | 0.87 | 0.57 | 1.33 | 1.14 | 0.78 | 1.67 |
| ≥7 | 1.00 |  |  | 1.00 | |  | | | |  | 1.00 |  |  | 1.00 |  |  |
| Environmental/other factors |  |  |  |  | |  | | | |  |  |  |  |  |  |  |
| Mode of delivery |  |  |  |  | |  | | | |  |  |  |  |  |  |  |
| Normal | 1.13 | 1.02 | 1.24 | 1.07 | | 0.97 | | | | 1.18 | 1.02 | 0.92 | 1.14 | 1.08 | 0.97 | 1.20 |
| Cesarean | 1.00 |  |  | 1.00 | |  | | | |  | 1.00 |  |  | 1.00 |  |  |
| Breastfeeding |  |  |  |  | |  | | | |  |  |  |  |  |  |  |
| No | 0.99 | 0.89 | 1.10 | 1.16** | | 0.88 | | | | 1.53 | 1.00** | 0.78 | 1.29 |  |  |  |
| Yes | 1.00 |  |  | 1.70 | | 1.28 | | | | 2.24 | 1.35 | 1.04 | 1.75 |  |  |  |
| Never breastfed |  |  |  | 1.00 | |  | | | |  | 1.00 |  |  |  |  |  |
| TV watching total |  |  |  |  |  | | |  | | |  |  |  |  |  |  |
| Less |  |  |  |  |  | | |  | | | 0.98 | 0.83 | 1.16 | 0.99 | 0.85 | 1.14 |
| more |  |  |  |  | | |  | |  | | 1.00 |  |  | 1.00 |  |  |
| TV at nighttime |  |  |  |  |  | | |  | | |  |  |  |  |  |  |
| Less |  |  |  |  | | |  | |  | | .99 | 0.86 | 1.16 | 0.96 | 0.85 | 1.08 |
| more |  |  |  |  | | |  | |  | | 1.00 |  |  | 1.00 |  |  |
| Younger children at home |  |  |  |  | | |  | |  | |  |  |  |  |  |  |
| No |  |  |  |  | | |  | |  | | 1.03 | 0.86 | 1.24 | 1.09 | 0.97 | 1.24 |
| Yes |  |  |  |  | | |  | |  | | 1 |  |  | 1 |  |  |
| Older children at home |  |  |  |  | | |  | |  | |  |  |  |  |  |  |
| No |  |  |  |  | | |  | |  | | 0.85 | 0.73 | 0.99 | 0.94 | 0.83 | 1.07 |
| Yes |  |  |  |  | | |  | |  | | 1 |  |  | 1 |  |  |
|  |  |  |  |  | | |  | |  | |  |  |  |  |  |  |

CI, confidence interval.

**p*<0.01, ***p*≤0.001.

Table S6. Poisson regression to examine the associations between sleep disturbances at 24 and 48 months and sociodemographic characteristics.

|  | **24 months *N*=2878** | | | | **48 months *N*=2974** | | | |  |
| --- | --- | --- | --- | --- | --- | --- | --- | --- | --- |
| Maternal characteristics |  | **B** | **95% CI** | |  |  | **B** | **95% CI** | |
| Maternal skin color |  |  |  |  |  |  |  |  | |
| White |  | 0.99 | 0.87**–**1.12 | |  |  | 0.99 | 0.87**–**1.13 | |
| Black |  | 1.04 | 0.91**–**1.20 | |  |  | 1.07 | 0.93**–**1.23 | |
| Other |  | 1^a^ |  |  |  |  | 1^a^ |  | |
| Maternal age (years) |  |  |  |  |  |  |  |  | |
| ≤19 |  | 0.96 | 0.84**–**1.10 | |  |  | 1.01 | 0.88**–**1.16 | |
| 20**–**34 |  | 1.04 | 0.94**–**1.15 | |  |  | 1.01 | 0.91**–**1.12 | |
| ≥35 |  | 1^a^ |  |  |  |  | 1^a^ |  | |
| Smoked during pregnancy |  |  |  |  |  |  |  |  | |
| No |  | 0.97 | 0.90**–**1.04 | |  |  | 1.03 | 0.95**–**1.11 | |
| Yes |  | 1^a^ |  |  |  |  | 1^a^ |  | |
| Alcohol during pregnancy |  |  |  |  |  |  |  |  | |
| No |  | 1.02 | 0.84**–**1.22 | |  |  | 1.03 | 0.85**–**1.25 | |
| Yes |  | 1^a^ |  |  |  |  | 1^a^ |  | |
| Maternal schooling (years) |  |  |  |  |  |  |  |  | |
| 0**–**4 |  | 1.10 | 0.99**–**1.23 | |  |  | 1.04 | 0.93**–**1.16 | |
| 5**–**8 |  | 1.05 | 0.97**–**1.14 | |  |  | 1.04 | 0.96**–**1.12 | |
| ≥9 |  | 1^a^ |  |  |  |  | 1^a^ |  | |
| Parity |  |  |  |  |  |  |  |  | |
| 1 |  | 1.04 | 0.93**–**1.17 | |  |  | 1.04 | 0.94**–**1.16 | |
| 2 |  | 1.01 | 0.92**–**1.11 | |  |  | 0.94 | 0.86**–**1.03 | |
| ≥3 |  | 1^a^ |  |  |  |  | 1^a^ |  | |
| Family income quintiles |  |  |  |  |  |  |  |  |  |
| 1 |  | 1.03 | 0.92**–**1.16 | |  |  | 0.96 | 0.85**–**1.08 | |
| 2 |  | 1.01 | 0.90**–**1.13 | |  |  | 0.95 | 0.85**–**1.07 | |
| 3 |  | 0.99 | 0.89**–**1.10 | |  |  | 1.04 | 0.93**–**1.16 | |
| 4 |  | 0.99 | 0.89**–**1.10 | |  |  | 0.95 | 0.85**–**1.06 | |
| 5 |  | 1^a^ |  |  |  |  | 1^a^ |  | |
| Child characteristics |  |  |  |  |  |  |  |  |  |
| Child gender |  |  |  |  |  |  |  |  |  |
| Male |  | 1.05 | 0.99**–**1.12 | |  |  | 1.01 | 0.95**–**1.08 | |
| Female |  | 1^a^ |  |  |  |  | 1^a^ |  | |
| Gestational age at birth (weeks) |  |  |  |  |  |  |  |  |  |
| ≤28 |  | 0.95 | 0.45**–**2.02 | |  |  | 0.95 | 0.47**–**1.93 | |
| 29**–**32 |  | 0.80 | 0.58**–**1.09 | |  |  | 0.83 | 0.62**–**1.11 | |
| 33**–**36 |  | 1.06 | 0.98**–**1.15 | |  |  | 1.04 | 0.96**–**1.13 | |
| ≥37 |  | 1^a^ |  |  |  |  | 1^a^ |  | |
| Birth weight (g) |  |  |  |  |  |  |  |  |  |
| <2500 |  | 0.96 | 0.84**–**1.09 | |  |  | 1.08 | 0.96**–**1.22 | |
| ≥2500 |  | 1^a^ |  |  |  |  | 1^a^ |  | |
| Neonatal complications (low APGAR score) |  |  |  |  |  |  |  |  |  |
| <7 |  | 1.12 | 0.88**–**1.42 | |  |  | 0.94 | 0.72-1.23 | |
| ≥7 |  | 1^a^ |  |  |  |  | 1^a^ |  | |
| Environmental/other factors |  |  |  |  |  |  |  |  |  |
| Mode of delivery |  |  |  |  |  |  |  |  |  |
| Normal |  | 0.99 | 0.93**–**1.06 | |  |  | 0.94 | 0.88**–**1.01 | |
| Cesarean |  |  |  | |  |  | 1^a^ |  | |
| Co-sleeping |  |  |  |  |  |  |  |  |  |
| No |  | 1.01 | 0.94**–**1.08 | |  |  | 1.02 | 0.95**–**1.09 | |
| Yes |  |  |  |  |  |  | 1^a^ |  | |
| Breastfeeding |  |  |  |  |  |  |  |  |  |
| Yes |  | 0.98 | 0.84**–**1.15 | |  |  |  |  |  |
| No |  | 0.94 | 0.80**–**1.11 | |  |  |  |  |  |
| Never |  | 1^a^ |  |  |  |  |  |  |  |
| TV watching total |  |  |  |  |  |  |  |  | |
| Less |  | 1.05 | 0.95**–**1.17 | |  |  | 0.99 | 0.90**–**1.08 | |
| more |  | 1^a^ |  |  |  |  | 1^a^ |  | |
| TV at nighttime |  |  |  |  |  |  |  |  |  |
| Less |  | 0.98 | 0.88**–**1.08 | |  |  | 0.88** | 0.82**–**0.95 | |
| more |  | 1^a^ |  |  |  |  | 1^a^ |  | |
| Younger children at home |  |  |  |  |  |  |  |  |  |
| No |  | 0.97 | 0.86**–**10.9 | |  |  | 1.01 | 0.93**–**1.09 | |
| Yes |  | 1^a^ |  |  |  |  | 1^a^ |  | |
| Older children at home |  |  |  |  |  |  |  |  |  |
| No |  | 1.13* | 1.03**–**1.23 | |  |  | 1.01 | 0.93**–**1.10 | |
| Yes |  | 1^a^ |  |  |  |  | 1^a^ |  | |

CI, confidence interval.

* *p*<0.01, ***p*≤0.001., ^a^ Reference group
